# Identifying behavior change techniques (BCTs) in responsive feeding interventions to prevent childhood obesity—A systematic review

**DOI:** 10.1111/obr.13857

**Published:** 2024-11-04

**Authors:** Gráinne Máire Áine Ní Eidhin, Karen Matvienko‐Sikar, Sarah Anne Redsell

**Affiliations:** ^1^ School of Public Health University College Cork Cork Ireland; ^2^ School of Health Sciences University of Nottingham Nottingham UK

**Keywords:** behavior change techniques, childhood obesity prevention, responsive feeding behaviors, systematic review

## Abstract

**Background:**

Complex interventions that include responsive infant feeding components can potentially prevent childhood obesity. To develop a replicable responsive feeding intervention, an understanding of the most effective behavior change techniques (BCTs) and theory is needed.

**Objective:**

To identify the BCTs and theories used in interventions with responsive feeding components for caregivers of children ≤2 years.

**Methods:**

PsycINFO, CINAHL, Cochrane Library, EMBASE, and MIDIRS were searched from inception to May 2023. Studies of obesity prevention interventions with a responsive feeding component were included. BCT Taxonomy Version 1 and Michie and Prestwich theory coding method were applied.

**Findings:**

Eighteen interventions were identified; the number of BCTs ranged from 3 to 11 (mean = 5.5). The most used BCTs were “Instruction on how to perform a behaviour” (17/18) and “Adding objects to the environment” (13/18), which were commonly used in the nine trials demonstrating higher responsive feeding behaviours and the four trials reporting reduced likelihood of overweight or obesity, or rapid weight gain. Fifteen trials reported use of theory.

**Conclusion:**

BCT use was low in interventions with responsive feeding components. BCTs are replicable; their use in interventions, alongside theory, will ensure that key determinants of responsive feeding behavior are included in future obesity prevention interventions.

## 1 INTRODUCTION

Obesity has reached epidemic levels in both developed and developing countries. For instance, in 2020, 2 billion people had overweight and 279 million people had obesity globally.^1^ Of these, 39 million children under the age of 5 experienced overweight or obesity in 2020.^1^Childhood overweight and obesity track to adulthood^2^ and are associated with increased risk of adverse health outcomes, including diabetes and cardiovascular disease,^1^ adverse mental health conditions,^1^ and early mortality.^3^

The development of childhood overweight and obesity is complex and has been attributed to environmental factors, lifestyle factors, and cultural factors.^2^ The first 1000 days of a child's life, from conception to 2 years of age, are particularly important in the etiology and prevention of overweight and obesity.^4^ Specific infant feeding practices are associated with greater risk of childhood overweight and obesity^5^, ^6^ during this time. These include feeding‐related factors, such as non‐initiation or reduced breastfeeding, adding cereal to formula milk bottles, increased early juice consumption,^5^, ^6^ greater restrictive and pressurizing feeding styles^5^, ^6^ and uninvolved, indulgent or highly protective parenting.^6^ Responsive infant feeding is protective against childhood obesity and is a feature of responsive caregiving, as a parent learns to recognize and understand a child's developmental cues and needs.^7^ In a responsive feeding approach, the caregiver responds in a timely and developmentally appropriate manner to the child's hunger and satiety related cues.^7^, ^8^ Appropriate responding can be enacted by feeding or offering food to an infant when they express signs of hunger and not offering/providing food when they indicate they are full. Inversely, non‐responsive feeding styles are characterized by a caregiver's lack of awareness, understanding, and appreciation of a child's needs and cues.^9^

Interventions have been developed and implemented to promote changes to responsive feeding behaviors with the aim of reducing the risk of childhood overweight and obesity. There is evidence that multi‐component responsive feeding‐focused obesity prevention interventions delivered by healthcare professionals (HCPs) result in healthier weight trajectories for infants than interventions without responsive feeding components.^10^ However, there is insufficient definitive evidence detailing which intervention components specifically work in obesity prevention during infancy and early childhood at a population or individual level. Individual‐level interventions frequently lack consideration of behavior change techniques (BCTs) and theory in their development and evaluation.

Understanding what theories underpin obesity prevention interventions is important because theoretically informed interventions are more likely to target key determinants of behavior change and therefore have an increased likelihood of beneficial outcomes.^11^, ^12^ Similarly, interventions informed by behavioral science frameworks demonstrate benefits for behavioral and health outcomes. For instance, the use of empirically and theoretically informed frameworks such as the Behavior Change Wheel^13^ and the related taxonomy of behavior change techniques (BCTs)^14^ provide robust approaches to both developing and examining behavior change interventions. BCTs are replicable, observable, and irreducible components designed to change behavior.^14^ They have been utilized in interventions across health areas to promote both the uptake of positive behaviors and cessation of negative behaviors^15^ and have been included and identified in some previously developed childhood obesity prevention interventions.^16^, ^17^ In combination with theoretical underpinnings, understanding what BCTs are used in childhood obesity prevention is needed to determine potential mechanisms of effect and guide future intervention development.

A recent review of BCT use in childhood obesity prevention interventions in children up to 12 years found that in 63 identified studies, the use and reporting of BCTs was highly varied.^18^ Similarly, in the context of infant‐feeding interventions, a previous review of BCTs and theory use in HCP‐delivered interventions to prevent obesity found varied use of BCTs, with “Instruction on how to perform the behaviour” the most frequently used BCT in reviewed interventions.^17^ This review also found that only 50% of studies used appropriate theory in the development of interventions^17^ and that there was poor integration between theory and BCTs in intervention development. These findings indicate the need for the development of such interventions to more appropriately consider the use of theory and BCTs in HCP‐delivered interventions. This previous review did not focus solely on responsive feeding interventions, however, which have demonstrated increased benefits for childhood obesity prevention.^10^ As such, BCTs and theories that are used and relevant for responsive feeding are unknown, and so it is important to examine which BCTs and theories have been used to date to better understand which approaches might work best.

The aim of this review is therefore to identify what BCTs and theories are used in responsive feeding interventions for caregivers of children ≤2 years of age to prevent childhood obesity. The review will also examine which BCTs may be most useful for inclusion in future obesity prevention interventions that include responsive feeding components.

## 2 MATERIALS AND METHODS

The review is reported in line with the Preferred Reporting Items for Systematic Review and Meta‐Analysis (PRISMA‐P) guidelines. The protocol is registered on the International Prospective Register of Systematic Reviews (PROSPERO; CRD420223126220).^19^

### 2.1 Search strategy

PsycINFO, CINAHL, Cochrane Library, EMBASE, and Maternity and Infant Care database (MIDIRS) were searched from inception to June 2022, with an updated search conducted in May 2023 using the following search strategy:

### 2.2 Inclusion and exclusion criteria

The inclusion and exclusion criteria are: (see Table 1):

**TABLE 1:** Inclusion and exclusion criteria.

Criteria	Inclusion	Exclusion	Patient/public, intervention, outcome
Sample	Caregivers (e.g., biological parent(s), adoptive parent(s), foster parent(s), grandparent(s), other familial primary guardian(s), non‐familial primary guardian(s)) of infants aged 2 years of age or younger. Economically developed countries (as indicated by the Organisation for Economic Co‐operation and Development [OECD])	Not caregivers of infants under the age of 2 years at the time of the intervention Aim of the responsive feeding intervention to maintain or increase child weight gain. Infants with medical conditions that affected feeding and/or growth (e.g., low birthweight infants or preterm infants) Caregivers or infants with visual impairments Non‐economically developed (non‐OECD) countries	*Participants*—child* OR infant OR infancy OR toddler* OR newborn [MeSH] OR baby OR babies OR child OR pediatric OR paediatric OR parent OR parental OR mother OR mum OR mom OR father OR dad OR caregiver
Exposure of interest	Responsive feeding components in an intervention or a responsive parenting intervention that included a feeding component, with the aim of preventing overweight and obesity.	Intervention that did not include a responsive feeding component or was not a responsive parenting intervention that included a feeding component that aim to prevent overweight and obesity.	*Intervention/exposure*—Responsive feed* OR responsive parenting OR hunger cue* OR satiety cue* OR responsive*.
Research type	Quantitative, mixed methods only where quantitative data is presented separate to qualitative data.	Qualitative, mixed method where qualitative data and quantitative data are not presented separately.	*Outcome* (*prevention of*)—obesity OR obese OR overweight OR weight OR BMI OR weight OR body mass index OR anthropometric OR adipos*. *Study design*—randomised controlled trial OR randomized controlled trial OR RCT OR case‐control OR quasi‐experiment*.
Language	English	Any language other than English	
Article type	Peer reviewed	Conference abstracts, unpublished theses, editorials, commentaries	

### 2.3 Screening

Following deduplication of identified papers, titles and abstracts of unique articles were independently screened by at least two reviewers (GNE, KMS, SR). Full‐text screening was then conducted independently by at least two reviewers (GNE, KMS, SR). Any disagreements were resolved by discussion and/or recourse to a third reviewer. For included full texts, a manual search was conducted for Supporting Information, and, for any included protocols, a search for full texts was conducted. All included trials that listed or provided supplemental material, such as the protocols and/or trial registrations, were downloaded and searched by GNE when the data was being extracted from full texts to ensure that any additional relevant information available was included in this review.

### 2.4 Data extraction

Using a standardized electronic data collection form, the following data were extracted: (1) author, (2) year of publication, (3) study characteristics (study design, sample size, country of origin), (4) participant characteristics (age, gender, ethnicity, caregiver socioeconomic status, caregiver education, caregiver marital status, infant birth weight, infant weight status, infant dietary requirements), (5) intervention details (verbatim intervention descriptions, focus of the intervention, what the intervention entailed, timing of intervention commencement, facilitation of the intervention), (6) duration of follow‐up (if applicable), (7) details on the control (sample number, details on usual care conditions), (8) outcome of the intervention (including outcome definition, if given, measurement tool(s) used and the measurement timing), (9) any numerical study findings (e.g., *p* values, confidence intervals, any confounders adjusted for, crude and adjusted estimates, magnitude and the direction of intervention effects). Data were extracted by one reviewer (GNE), with 20% of extraction checked by a second reviewer (KMS, SR).

### 2.5 BCT coding

The Behaviour Change Technique Taxonomy version 1 (BCTTv1) was used to code for BCTs in reviewed interventions. The BCTTv1 is a taxonomy containing standardized terminology relating to 93 unique BCTs, which are categorized into 16 groups.^14^ The coding process was piloted on interventions and active control conditions reported in two random studies included in the review. As there were no issues in the pilot coding, all interventions and active control conditions in the included published articles and any related supplementary documentation, including protocols or intervention materials, were subsequently coded. Each BCT was coded as “+” if present and “−” if not present in the intervention. When it was unclear whether a BCT was present or absent, it was coded as “−”, as per BCTTv1 guidance.^14^ Usual care conditions were not coded, as these conditions were not added to the study with the intention of effecting the targeted behavior. Each study was coded by one reviewer (GNE); two other reviewers with training and experience in BCT coding double‐coded half of the studies each (KMS, SR).

### 2.6 Theory coding

The Theory Coding Scheme Michie and Prestwich^20^ was used to review the interventions. This coding scheme contains 19 items covering a range of areas in which theory can be incorporated into intervention design and evaluation. The coding has a clear description of how to code each item. Items 1–12 of this coding scheme were used to examine the extent to which the development and evaluation of the included interventions were theoretically based. Items 13–19 were not relevant for this review as they assess methodological issues related to measurement. One reviewer (SR) theoretically coded the interventions from their descriptions in the published literature, with a further reviewer (KMS) checking all scoring. Discrepancies between the reviewers were resolved by discussion. Three composite scores were calculated to evaluate theory use. The first composite score involved Items 7, 8, and 9 and was linked to a theory‐relevant construct. The second score (Items 9, 10, 11) assessed the extent to which theoretical constructs specifically targeted BCTs. The overall theory score was calculated by summarizing individual items related to the use of theory to develop the interventions, namely, Items 3, 4, 5, and 6, and adding this to the previous scores. The total score ranges from 0 (no theory use) to 8 (good use of theory).

### 2.7 Quality appraisal

All papers included in this systematic review were appraised using the Cochrane Risk of Bias tool.^21^ The most recent version of the Cochrane Risk of Bias tool is the recommended tool to assess the risk of bias for systematic reviews.^21^ Each article was independently assessed for risk of bias by two of the three reviewers (GNE, KMS, SR).

### 2.8 Data synthesis

To analyze the use of BCTs in the interventions included in this review, the total number of individual BCTs, percentage, and combinations of BCTs were calculated and presented in a tabular format. The BCTs and their relationship, if any, to intervention effects, such as an increased or a reduced effectiveness of interventions, were narratively synthesized. Only outcomes related to responsive feeding were reported; statistical examination of the relationship between BCTs and intervention effects was not deemed appropriate due to the heterogeneity in interventions, outcomes, and BCTS.

## 3 RESULTS

**FIGURE 1:**
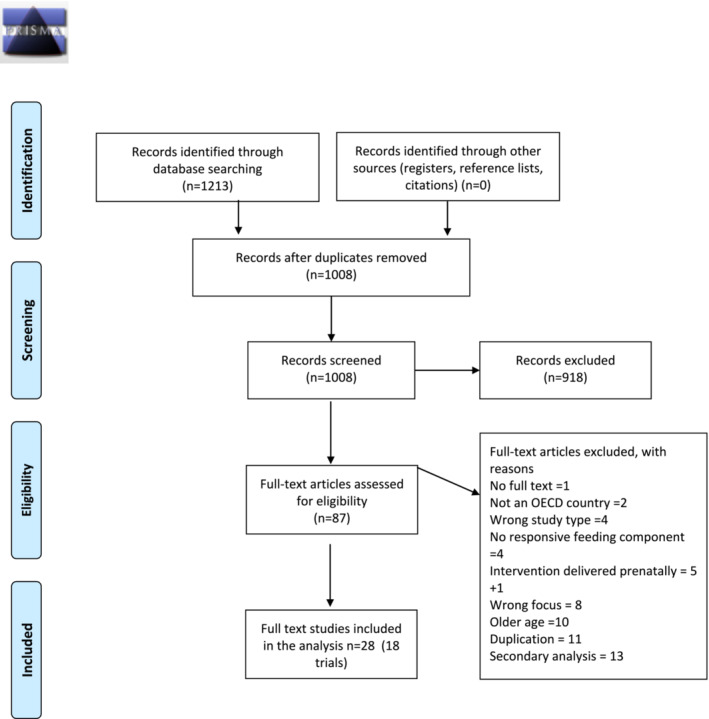
Preferred Reporting Items for Systematic Review and Meta‐Analysis (PRISMA) flow diagram.

The first comprehensive database search (from inception to June 2022) identified 1191 papers. Of these, 26 papers reporting on 16 trials were eligible for inclusion in this review. The second search in May 2023 identified 22 papers published from June 2022 onwards, of which two were eligible for inclusion, resulting in 28 papers reporting outcomes from 18 trials (see Figure 1 PRISMA flow diagram).

### 3.1 Trial characteristics

Of the 18 trials included, only one was not a randomized controlled trial.^22^ Five papers were RCT protocols only, without any reported outcomes at the time of this database search.^23^, ^24^, ^25^, ^26^, ^27^, ^28^ The majority of interventions (*n* = 12) were conducted in the United States of America (USA). These were: Educational intervention to modify feeding behaviors,^29^ FSN,^30^ Healthy Babies Trial,^27^ Home and Videotape Intervention,^31^ INSIGHT,^32^, ^33^, ^34^, ^35^ Mothers and Others,^36^, ^37^ PSE,^38^ SMS,^22^ SAAF,^24^, ^39^ The Baby Act Trial,^25^ Tools for Teen Moms,^28^ and WEE Baby.^26^ In addition, one intervention (NOURISH) was conducted in Australia,^40^, ^41^, ^42^ one was conducted in New Zealand (BLISS),^43^ and one was conducted in the United Kingdom (The Baby Milk Trial).^44^, ^45^ The remaining three interventions were conducted in Europe, with one conducted in the Netherlands (Baby's First Bites),^46^, ^47^ one in Norway (Sapere‐sensory education),^23^ and one in Italy (Probit).^48^ The number of participants in the included trials ranged from 41^29^ to 430^37^ (see Table 2 trial characteristics).

**TABLE 2:** Trial characteristics (*n* = 18).

Study	Study design	Study aim	Country and participants characteristics	Intervention	Control	Trial findings	Risk of bias
Baby's First Bites (BFB) (van der Veek^46^; van Vliet^47^)	RCT	To examine the effect of an intervention focusing on vegetable consumption (what) and maternal sensitivity during mealtimes (how) on weight and infant feeding outcomes	The Netherlands Mothers (first‐time) Aged 31 years (SD = 4.7) Almost 20% had a master degree Infant age at baseline: 4–6 months	Three intervention conditions *n* = 61 (RVE) + 62 (VIPP‐FI) + 60 (COMBI) RVE—focused on what the infant was being fed VIPP‐FI—focused on how the infant was being fed COMBI—consisted of components of both interventions mentioned above. Five intervention sessions delivered by researchers, individually, virtually, and in person Duration: 12 months Outcomes measurement at 18, 24, and 36 months.	*n* = 63 Consisted of five phone calls. Topics such as sleep, language and motor development, and complementary feeding experience were discussed.	*Primary outcomes* Child vegetable intake—no significant difference between the RVE, VIPP‐FI, and COMBI compared with the AC (*p* = 0.11–0.86). Significant increase in vegetable intake across all groups between the beginning of the intervention and the 24‐month follow up (*p* < 0.001). No differences in infant self‐regulation of energy intake between groups. *Secondary outcome* No significant difference in the BMI z‐scored between the groups at 12, 18, and 24 months.	Moderate risk
Baby‐Led Introduction to SolidS (BLISS) (Taylor et al.^43^)	RCT	To examine the effect of a baby‐led approach to complementary feeding compared with traditional spoon‐feeding on weight and infant feeding outcomes	New Zealand Mothers (<40% first‐time) Aged 31.3 years (SD = 5.0–6.20) 71.3% attended third level Over 40% employed full time—“relatively socioeconomically advantaged” Infant age at baseline: 1 week	*n* = 105 Focused on extending exclusive breastfeeding and delaying the introduction of complementary feeding until 6 months of age Participants receive eight supplementary contacts from a lactation consultant (five) and trained researcher (three) from pregnancy to 9 months of age Delivered by lactation consultant and trained researcher, individually, in person and virtually. Duration: 9 months. Outcomes at 6, 7, 8, 9, 12, and 24 months. Weight at 12 and 24 months	*n* = 101 Focused on usual care. Delivered by healthcare professionals, including a midwife. Participants had access to standard government‐funded midwifery and well‐childcare.	*Primary outcome* No significant difference in infant BMI score between the intervention and control groups at 12 and 24 months. *Secondary outcomes* Mothers of BLISS infants rated their infants as significantly less fussy or picky about food, whether measured by the CEBQ (adjusted difference, −0.33; 95% CI, −0.51 to −0.14) or TMBQ (adjusted difference, −0.22; 95% CI, −0.41 to −0.04) at 12 but not 24 months of age Intervention infants made significantly more mealtime decisions than control infants at 12 months (adjusted difference, 0.16; 95% CI, 0.01–0.31)	Moderate risk
Educational Intervention to Modify Bottle‐feeding Behaviors among Formula‐feeding Mothers in the WIC Program (Kavanagh et al.^29^)	RCT	To examine the impact of promoting awareness of satiety cues and discouraging bottles on formula feeding practices and weight outcomes.	Women, infants, and children (WIC) clinics in US intervention caregivers aged 24.7 years (SD 4.9), control 26.1 (4.5) Infant age 3–10 weeks. Formula fed only	*n* = 19 (formula intake) n = 18 weight One 45–60‐min class delivered by educators. Leaflet “Happiest Baby on the Block.” Outcomes at: 4–5 months (formula intake and growth)	*n* = 21 (formula intake) *n* = 20 weight. Classes on best practices for complementary feeding (both groups)	*Primary outcome* No significant difference in formula feeding intake between the two groups *Secondary outcome* Infant growth in the intervention group was greater than in the control group (*p* < 0.01) contrary to the hypothesis.	Moderate risk
Family Spirit Nature (FSN) (Rosenstock et al.^30^)	RCT	To evaluate the effect of a home visiting intervention on responsive parenting, infant feeding practices and weight outcomes	USA Mothers aged 27.4 years (SD = 6.2) Native American Most attended third level 45% had no financial hardship Infant age at baseline: 2 months	*n* = 68 Focused on encouraging responsive/complementary feeding practices and avoiding sugar sweetened beverages. Participants received six home visits which discussed topics such as the importance of eating healthy among the whole family, controlling sugar intake, infant's hunger/satiety cues, delaying the introduction of solid foods until 6 months post‐partum, breastfeeding, mother and infant bonding, traditional Native American diet delivered by Navajo paraprofessionals, individually, in person. Duration: 3 months Outcomes measurement at 4, 6, 7, 8, 9, and 12 months. Weight only at 12 months	*n* = 66 Focused on injury prevention. Delivered by Navajo paraprofessionals, individually, in person Duration: 3 months Participants received injury prevention education at three one‐on‐one lessons.	*Primary outcomes* Responsive feeding behaviors were significantly higher among the intervention group than the control group at 6 months (3.54 vs. 3.29, *p* = 0.07) and 9 months (3.48 vs. 3.22, *p* = 0.07) but not at 12 months Infants in the intervention group were fed their first foods an average of 3 weeks earlier than the control group. Sugar sweetened beverage consumption was significantly lower among infants in the intervention group than the infants in the control group at 9 months of age (mean 0.09 cups per vs. 0.43 cups per week) and at 12 months of age (mean 0.56 cups per week vs. 1.78 cups per week) *Secondary outcome* BMI was significantly lower among intervention infants than among control infants at 6 months (0.16 vs. 0.63) and at 9 months (0.27 vs. 0.81) but not at 12 months.	High risk
Healthy Babies Trial (Horodynski et al.^27^)	RCT protocol	To compare the effect of a home‐based intervention for economically and educationally disadvantaged mothers of infants 4 months of age or younger versus the standard nutrition education on maternal responsiveness, feeding style, and feeding practices.	USA Mothers (first‐time) Low‐income Educationally disadvantaged Infant age at baseline: 6 months	*n* = 186 (calculated sample size) Focused on the development of healthy infant eating practices. Six in‐home lessons (60 min each) followed by three reinforcement telephone calls (5 min each). Lessons include opportunities for mothers to develop/practice skills, discussions about strategies to overcome challenges/problem‐solving techniques, and animated DVD to demonstrate infant temperament traits. Calls used to reinforce key concepts and maintain effects/sustain. Delivered by trained paraprofessional instructor, individually, in person and virtually. Duration: ≤12 months Outcomes measurement at 6 and 12 months	*n* = 186 (calculated sample size) Focused on usual care. Delivered by paraprofessional instructor, individually, in person. Six in‐home lessons (60 min each). Lessons consists of information on nutrition and the major food groups.	/	Moderate risk
Home and Videotape Intervention (Black et al.^31^)	RCT	To assess the effectiveness of an intervention that aims to delay the early introduction of complementary feeding among this population (first‐time, Black, adolescent mothers living in multigenerational households)	USA Mothers (first‐time) Aged 16.4 years (SD = 0.9) 100% African American Education: Grade 7–12 Low‐income households Infant age at baseline: 4–6 weeks	*n* = 58 Focused on decreasing the cultural obstacles to the acceptance of recommendations regarding complementary feeding practices. Fortnightly home visits were conducted until infant was 12 months old (22–24 home visits). Delivered by research assistant, individually, in person, and virtually. Duration: 12 months Outcomes measurement at 3 months of age Mothers were contacted via phone to complete a FFQ for infant's dietary intake. No measurements recorded beyond infant age 3 months. No weight outcomes collected	*n* = 63 Focused on usual care Follow‐up was the same as intervention follow‐up—FFQ for infant diet.	*Primary outcome* At three mothers of infants in the optimal feeders group were more likely to report accurate messages regarding the timing to begin complementary feeding (*p* = 0.005) and almost four times more likely to be in the intervention group than the control group (*p* = 0.005).	Low risk
Intervention Nurses Start Infants Growing on Healthy Trajectories (INSIGHT) (Paul et al.^35^; Savage et al.^32^, ^34^; Adams et al.^33^)	RCT	To determine whether a responsive parenting intervention affects infant feeding behaviors	USA Mothers (first‐time) Mean age = 28.6 years (SD = 4.4–4.9) >90% White >90% attended college >65% higher income Infant age at baseline: 3–4 weeks	*n* = 140 Focused on responsive parenting (RP) in the four behavioral states (drowsy, sleepy, fussy, and alert/calm). At the visits, guidance on infant feeding under three headings, “what to feed,” “when to feed,” and “how to feed,” was given to mothers. Delivered by trained research nurses, individually, in person. Duration: Four home visits at 3–4, 16‐, 28‐, and 40‐weeks post‐partum and one research center visit at 1 year. Outcomes measurement from 2 weeks to 36 months.	*n* = 139 Focused on usual care. Delivered by trained research nurses, individually, in person. Consisted of five home visits at 3–4, 16‐, 28‐, and 40‐weeks post‐partum and one research center visit at 1 year. Lessons on home safety guidance.	*Primary outcome* RP infants gained weight more slowly than control group infants (0.18; 95% CI, 0.02–0.34); this effect did not differ by feeding mode (predominantly fed breast milk or not). Infants in the RP group had lower mean weight‐for‐length percentiles at 1 year than infants in the control group (57.5%; 95% CI, 52.56%–62.37% vs. 64.4%; 95% CI, 59.94%–69.26%; *p* = 0.04) and were less likely to be overweight at age 1 year (5.5% vs. 12.7%; *p* = 0.05) *Secondary outcomes* RP mothers were more likely to use of structure‐based feeding practices including limit‐setting (*p* < 0.05) and consistent feeding routines (*p* < 0.01) at age 1 year. RP group mothers were less likely to use non‐responsive feeding practices such as pressuring their infant to finish the bottle/food (*p* < 0.001), and using food to soothe (*p* < 0.01), propping the bottle (*p* < 0.05) assessed between 4 and 8 months, and putting baby to bed with a bottle at age 1 year (*p* < 0.05). RP children had less daily screen time than controls at each time point (*p* ≤ 0.01) and less television during infant meals (*p* < 0.05).	Low risk
Mothers and Others (Wasser et al.^36^, ^37^)	RCT	To test the effectiveness of a home‐based intervention on infant size and growth	USA Non‐Hispanic Black women and partners Age 25.8 years (SD = 5.3) Single (72.2%) Receiving Medicaid (74.4%)	*n* = 213 at baseline, 122 at infant age 3 months, 113 at 9 months, 104 at 15 months. Anticipatory guidance on breastfeeding, responsive feeding, use of non‐food soothing techniques for infant crying, appropriate timing and quality of complementary feeding, age‐appropriate infant sleep, and minimization of TV/media. Delivered by peer educator six home visits four interim newsletters and twice‐weekly text messaging. Duration: 28 and 37 weeks gestation and when infants are 1, 3, 6, 9, 12, and 15 months. Outcomes measurement at 15 months	*n* = 214 at baseline, 130 at infant age 3 months, 111 at 9 months, 99 at 15 months. Injury Prevention Group (IPG) Attention‐control group on child safety also consisting of PE‐delivered home visits, newsletters, reinforcing text messages	*Primary outcome* No significant difference in weight‐for‐age z‐score (WAZ) (WAZdiff = −0.07, 95% CI: −0.40–0.25, *p* = 0.659 ). *Secondary outcomes* No responsive feeding outcomes reported in included papers.	Moderate risk
NOURISH (Daniels et al.^40^, ^41^, ^42^)	RCT	To examine the effect of a obesity prevention intervention on weight and infant feeding outcomes	Australia Mothers (first‐time) Aged 30.1 years (SD = 5.3) 78% Australia/New Zealand born 58% had university education 33% score as relative disadvantage Infant age at baseline: 4–7 months	*n* = 352 at baseline, 291 at 14 months, 260 at 18 months Focused on feeding and parenting practices. Two skill‐based modules (delivered at 4–7 months and 13–26 months) of six fortnightly in person group sessions, each of 1–1.5 h duration. Two themes in each module—(1) repeated neutral exposure to unfamiliar foods and limiting exposure to unhealthy foods to promote the development of preferences towards healthy foods. (2) responsive feeding that recognizes and responds appropriately to infant hunger and satiety cues to maintain infants' instinctive capacity to self‐regulate intake and avoid overfeeding Delivered by a dietitian and a psychologist, in a group and in person. Duration: 12 months Outcomes at 14, 18, and 24 months of age, and twice after 24 months of age.	*n* = 346 at baseline, 307 at 14 months, 281 at 18 months Focused on usual care—existing issues, not anticipatory guidance. Delivered individually, both in person and virtually. Self‐directed access to usual community child health services.	*Primary outcome* Intervention infants had a lower average BMI than control infants (*p* = 0.009) and were less likely to display rapid weight gain than control infants (*p* = 0.014) at 14 months. Intervention effects were not sustained at 2–5 year follow up. *Secondary outcomes* At 14 months, there were significant intervention effects for hunger and satiety cues (*p* = 0.007). Mothers in the intervention group appeared to be more persistent in reoffering new foods (*p* < 0.01) and less likely to disguise new foods (*p* < 0.01). Mothers from the intervention group reported less frequent use of 2/5 strategies (*p* < 0.01) that override child satiety signals and more frequent use of strategies (*p* = 0.07) that respond appropriately to these signals. Significantly less non responsive feeding behaviors were observed in intervention mothers at child aged 2, 3.7, and 5 years.(*p* < 0.05). Mothers in the intervention group were less likely to disguise the food and more likely to continue to reoffer new foods (two of four items).	Moderate risk
Policy, System and Environmental (PSE) strategies for promoting responsive bottle‐feeding practices (Ventura et al.^38^)	Cluster RCT	To examine the effectiveness of PSE strategies for encouraging responsive bottle‐feeding within the WIC program on weight and infant feeding outcomes	USA Mothers (<60% first‐time) Aged 28.7 (SD = 6.2) Almost 60% attended some form of third level education Low‐income Infant age at baseline: <60 days	*n* = 124 Focused on encouraging responsive bottle feeding. Two PSE strategies were implemented in three WIC clinics. The first PSE strategy involved altering the “Assessment of Early Infant Feeding Decisions” The second strategy involved developing educational materials to encourage staff discussions about responsive bottle‐feeding. Delivered by WIC clinic staff, individually, in person. Duration: up to 6 months infant age. Outcomes measurement at 3 and 6 months infant age.	*n* = 122 Focused on usual care. Delivered by WIC clinic staff. Duration: 6 months The PSE strategies were not implemented in the control clinics.	*Primary outcome* Infants in intervention group were at a lower risk of rapid weight gain than infants in the control groups at 4–6 months of age (*p* = 0.014). *Secondary outcomes* Mothers in the intervention group and the control group exhibited similar levels of responsive feeding and pressuring feeding. Mothers in intervention groups reported feeling more supported about their decision to bottle feed (*p* = 0.098) and had stronger intentions of continuing with the WIC program (*p* = 0.002).	High risk
Prevention of Obesity in Toddlers (PROBIT) (Morandi et al.^48^)	Probit RCT	To examine the impact of an infant feeding intervention on weight and infant feeding outcomes	Italy Mothers (not reported) Infant age at baseline: 1 month No other information stated	*n* = 295 at baseline, 252 at follow‐up Focused on encouraging protective feeding related behaviors and discouraging destructive feeding related behaviors. Parents provided with standardized oral and written information concerning protective practices at all five routine visits scheduled during the children's first 2 years of life (at 1, 3, 6, 12, and 24 months of age). Parents provided with educational leaflet Delivered by trained pediatrician, individually, and in person. Duration: 24 months Outcomes measurement at 24 months.	*n* = 267 at baseline, 216 at follow‐up Focused on usual care Delivered by “control pediatricians,” individually and in person. Infants provide usual care and follow‐up at the routine visits.	*Primary outcome* No significant difference in BMI between intervention and control groups at 3, 6, and 12 months. Prevalence of obesity among the 2‐year‐old infants in the intervention group was 35% lower than among the controls group (*p* = 0.10). *Secondary outcome* A higher percentage of infants from the intervention group were being fed responsively than infants in the control group at 3 months of age (93% vs. 80%) (*p* < 0.001). No significant difference in feeding outcomes at 6 or 12 months.	High risk
Sapere‐sensory education and healthy meal intervention (Helland et al.^23^)	Cluster RCT protocol	To examine the impact of an intervention designed to promote healthy feeding practices and reduce levels of food neophobia	Norway Kindergarten staff and parents (not reported) Infant age at baseline: 2 years	*n* = 43 kindergarten staff and 69 children Focused on reducing food neophobia in toddlers and promoting healthy feeding practices among kindergarten staff and parents. Kindergarten staff introduced infants to a new vegetable—three times a week, different state each time (raw, mashed, pickled, etc.) using toys and prompts. The infants also received healthy lunch options at the kindergarten. Parents received information on the intervention. Delivered by Helland and trained kindergarten staff, in person, group. Duration: 9 weeks. Outcome measurement, up to 4 years of age.	*n* = 39 kindergarten staff and 47 children Focused on usual practices. Participants received their usual pedagogical sessions, meals, and food serving practices. The parents did not receive any information.	/	Low risk
Short Message Service (SMS) (Palacios et al.^22^)	Feasibility trial	To examine the impact of weekly SMS for improving infant feeding practices and infant weight status	USA, Puerto Rico, and Hawaii 99.5% mothers (not reported) Aged 27 (SD = 5.02–5.27) Most Hispanic and White Over 40% did not attend third level Low‐income Infant age at baseline: 0–2 months	*n* = 84 Focused on encouraging breastfeeding, preventing overfeeding, reducing juice consumption, and prolonging the introduction of solid foods. One message was delivered to mothers each week. The messages were 35–50 words long and included information on why behaviors should be performed and advice on how to perform the behavior. To identify if mothers were reading the text messages, seven short questions about the content of the weekly texts were sent via text. The mothers response to these questions and the replies were recorded. Delivered virtually based on WIC recommendations. Duration: 4 months Outcomes measurement at 4 months.	*n* = 86 Focused on usual care. Delivered virtually based on WIC recommendations. Duration: 4 months SMS about infant's general health issues, including bathing, sleeping, and teething.	*Primary outcomes* No differences between the intervention and control groups, which included exclusive breastfeeding rates, the age of introduction to complementary feeding, rates of placing the infants to sleep with milk bottles, the caregiver's method and response to infant feeding. Higher percentage of caregivers in the intervention group stopped feeding when infants displayed signs of being full (44.1%) compared with controls (39.5%) (*p* = 0.07). *Secondary outcome* No significant group differences in weight from Visit 1 to Visit 2 (4 months later).	Moderate risk
Sleep strong African American families (SAAF) (Lavner et al.^24^; Hernandez et al.^39^)	RCT protocol—recruitment stage is completed	To evaluate the impact of responsive parenting (focused on infant sleep and soothing) on decreasing rapid weight gain and parental and infant behaviors	USA Mothers (first‐time) African American Infant age at baseline: 3 weeks	*n* = / Focused on shaping parenting behaviors related to sleep and soothing, appropriate, and responsive feeding, and routines to prevent early obesity. Two home visits to provide responsive parenting information to the mothers on four areas (sleeping, crying, feeding, and playing) at 1, 3, 8, and 16 weeks. Delivered by community research associates, individually and in person. Duration: 16 weeks Outcome measures: 3–16 weeks.	n = / Focused on general baby safety. Delivered by community research associates Duration: 16 weeks Two home visits. Topics of general baby safety discussed	*Primary outcome* Weight was reported as primary outcome in protocol but not in final paper. *Secondary outcomes* RP mothers reported more responsive feeding (*p* = 0.005, lower likelihood of using beverages other than breast milk/formula to soothe their infant (*p* = 0.01) and less pressure with cereal than control mothers (*p* = 0.09) RP mothers also reported less pressure to finish/soothe than controls (*p* = 0.007, feeding mode (*p* = 0.003) and maternal age (0.04) moderated this effect.	Moderate risk
The Baby Act Trial (Campos et al.^25^)	Cluster RCT protocol—recruiting stage	To identify the impact of an infant feeding intervention on healthy weight gain and health related behaviors (physical activity, sleep, and food intake) compared with usual care practices.	USA–Puerto Rico Mothers (not reported) Aged 18+ Hispanic Low‐income Infant age at baseline: right after birth	n = / Focused on age‐appropriate infant physical activity, healthy sleep and sedentary patterns, and responsive feeding. Consisted of weekly web‐based lessons and text messages, and phone calls every 3 months. Educational videos to encourage age‐appropriate behaviors relating to physical activity. Information on screen time and responsive feeding was also provided in the lessons. Delivered by dietitians, individually, virtually. Duration: 12 months Outcome measurement at 12 months	n = / Focused on usual care. Delivered by healthcare professionals, individually and group, online and in person. Consists of standardized home visits at 0–1, 6, and 12 months. Also consists of two educational contacts every 6 months—online or in person.	/	
The Baby Milk Trial (Lakshman et al.^44^, ^45^)	RCT	To examine the impact of an intervention designed to reduce formula‐milk intake, encourage responsive feeding and healthy weaning, and avoid excessive weight gain	UK Mother (<50% first‐time) Aged 31.7 (SD = 5.5–5.9) Almost 40% had a degree or higher. ~90% employed >90% White >55% married Infant age at baseline: 2–3 months	*n* = 340 Focused on reducing formula milk intake and promoting responsive feeding practices. Consisted of three in person visits (30–45 mins long) and two phone calls (15–20 min long). Feeding recommendations were discussed, including infant nutrition, how to prepare formula milk and weaning advice. Delivered by trained facilitators (research nurses) individually, in person and virtually. Duration: 4 months. Outcome measurement at 3,4, 5, 12 months. Weight at 6 and 12 months.	*n* = 329 Focused on usual care. Duration: 4 months The control group were offered the same number of home visits and phone calls. General information regarding formula feeding was discussed, including the sterilization of feeding equipment.	*Primary outcome* No significant effect on weight gain to 12 months. *Secondary outcomes* Mothers in the intervention group reported a strengthened maternal attitudes to following the infant feeding recommendations. Infants in the intervention group had reduced milk intake at 3 months (−14% intervention infants vs. control infants), at 4 months (−12%), at 5 months (−9%), and at 6 months (−7%).	Moderate risk
Tools for Teen Moms (Horodynski et al.^28^)	RCT protocol	To examine the impact of a social media intervention on infant weight, maternal responsiveness, and feeding style and practices	USA Mothers (first‐time) Aged 15–19 years African Americans Low‐income (≤185% federal poverty level) Infant age at baseline: 1–2 months	n = Focused on aiding teenage mothers in reducing the risk of infant obesity utilizing technology. Consists of usual care (see control) plus daily text messages prompting the use of the T4TM web‐based application and to complete the behavioral The daily challenges target topics such as maternal responsiveness, infant temperament, feeding style and practices, and feeding environment. Delivered by registered nurse, licensed social worker, registered dietician, infant mental health specialist and/or paraprofessional, individually, in person and virtually. Duration: 6 weeks (up to 1 year usual care). Outcome measurement at 4–8 weeks, 10–12 weeks, and 6 months.	*n* = Focused on usual care. Delivered by registered nurse, licensed social worker, registered dietician, infant mental health specialist and/or paraprofessional, individually, in person. Duration: up to 9 months Consists of voluntary home visits (at 1, 6, and 24 weeks postpartum) and continued visits (as needed—max nine visits up to 1 year) based on the participants' personalized care plan following in‐home assessments.	/	Low risk
Wee Baby Care (Savage et al.^26^)	RCT protocol	To examine the effect of a responsive parenting intervention on rapid infant weight and parenting behaviors	USA Mothers (first‐time) Aged 18–55 years Low income Infant age at baseline: 2 months	n = / Focused on coordinating care between clinical and community settings to improve messaging between providers, to improve parent self‐efficacy, and shared parent–child decision. Delivered by pediatric primary care providers and nutritionists, individual and in person. Duration: 6 months The educational aspect included information feeding, soothing, sleeping, and playing. The regularly scheduled include five well‐care visits and three visits with a nutritionist.	n = / Focused on usual care. Delivered by pediatric primary care providers and nutritionists. Duration: Participants receive standard care.	/	Unclear risk

Abbreviations: AC, active control; SD, standard deviation; RCT, randomized control trial; PSE, policy, systems, and environmental; RVE, repeated vegetable exposure intervention; VIPP‐FI, video intervention to promote positive parenting–feeding infants intervention; COMBI, combined condition of RVE and VIPP‐FI.

### 3.2 Intervention characteristics

As reported in Table 2, the intervention facilitator varied between the interventions, with seven interventions delivered by HCPs only. These were INSIGHT,^32^, ^33^, ^34^, ^35^ NOURISH,^40^, ^41^, ^42^ PSE,^38^ (Probit),^48^ The Baby Act Trial,^25^ (The Baby Milk Trial),^44^, ^45^ Tools for Teen Moms,^28^ and WEE Baby^26^. The BLISS intervention was delivered by both a researcher and a HCP^43^; FSN was delivered by paraprofessionals (a US term for educators)^30^; the Mothers and Others intervention was delivered by a peer educator.^36^, ^37^ The Home and Videotape Intervention,^31^ SAAF,^24^, ^39^ and (Baby's First Bites)^46^, ^47^ were delivered by researchers only. The Sapere‐sensory education intervention was delivered by a researcher and kindergarten staff.^23^ The Educational intervention to modify feeding behaviors^29^study reported their intervention was delivered by “trained educators” but did not specify their background. The SMS study did not report the type of intervention facilitator.^22^ Intervention durations ranged from 6 weeks^26^, ^38^ to 2 years,^48^ with an average of 9 months. The majority of the interventions in this review included six or more lessons or contacts with the facilitator.^22^, ^25^, ^30^, ^31^, ^32^, ^33^, ^34^, ^35^, ^36^, ^37^, ^40^, ^41^, ^42^, ^43^ Frequency of delivery of intervention components varied between interventions, and ranged from two visits to mothers and infants delivered by African American Community Research Associates at 3+ and 8 weeks post‐partum for SAAF^24^, ^39^ to two skill based modules with six lessons in each of 1–1.5 h for NOURISH.^40^, ^41^, ^42^ Intervention delivery method also varied: nine interventions were delivered in person^23^, ^24^, ^26^, ^29^, ^30^, ^32^, ^33^, ^34^, ^35^, ^38^, ^39^, ^40^, ^41^, ^42^, ^48^; two were delivered virtually^22^, ^25^; and seven were delivered both in person and virtually.^25^, ^27^, ^28^, ^31^, ^43^, ^44^, ^45^, ^46^, ^47^ Thirteen interventions were delivered to participants individually^24^, ^25^, ^26^, ^27^, ^28^, ^30^, ^31^, ^32^, ^33^, ^34^, ^35^, ^36^, ^37^, ^39^, ^43^, ^44^, ^45^, ^46^, ^47^, ^48^ and three interventions were delivered in a group setting.^23^, ^29^, ^40^, ^41^, ^42^ Eight interventions were delivered in participants homes,^24^, ^27^, ^28^, ^30^, ^31^, ^32^, ^33^, ^34^, ^35^, ^36^, ^37^, ^39^, ^44^, ^45^ five interventions were delivered in a clinical setting^26^, ^38^, ^40^, ^41^, ^42^, ^44^, ^45^, ^48^ one intervention was delivered in both the home and a research center,^32^, ^33^, ^34^, ^35^ and one intervention was delivered in a kindergarten.^23^

### 3.3 Risk of bias assessment

Overall, the risk of bias quality assessment indicated low to moderate risk of bias across included studies.^21^ The areas in which the studies were at the highest risk of bias were performance bias and reporting bias. Of the studies included in this review, 72% (13 out of 18 trials) had a high risk of performance bias due to participant and facilitator awareness of the intervention allocation. Sixty‐six percent of trials (*n* = 12) were rated as high risk of performance bias, and two trials had an unclear risk of reporting bias.^29^, ^31^ In addition, 22% of trials (*n* = 6) had a high risk of detection bias due to a lack of blinding of the outcome assessment^24^, ^30^, ^32^, ^33^, ^34^, ^35^, ^36^, ^37^, ^39^, ^48^ (see Table 3 risk of bias).

**TABLE 3:** Risk of bias.

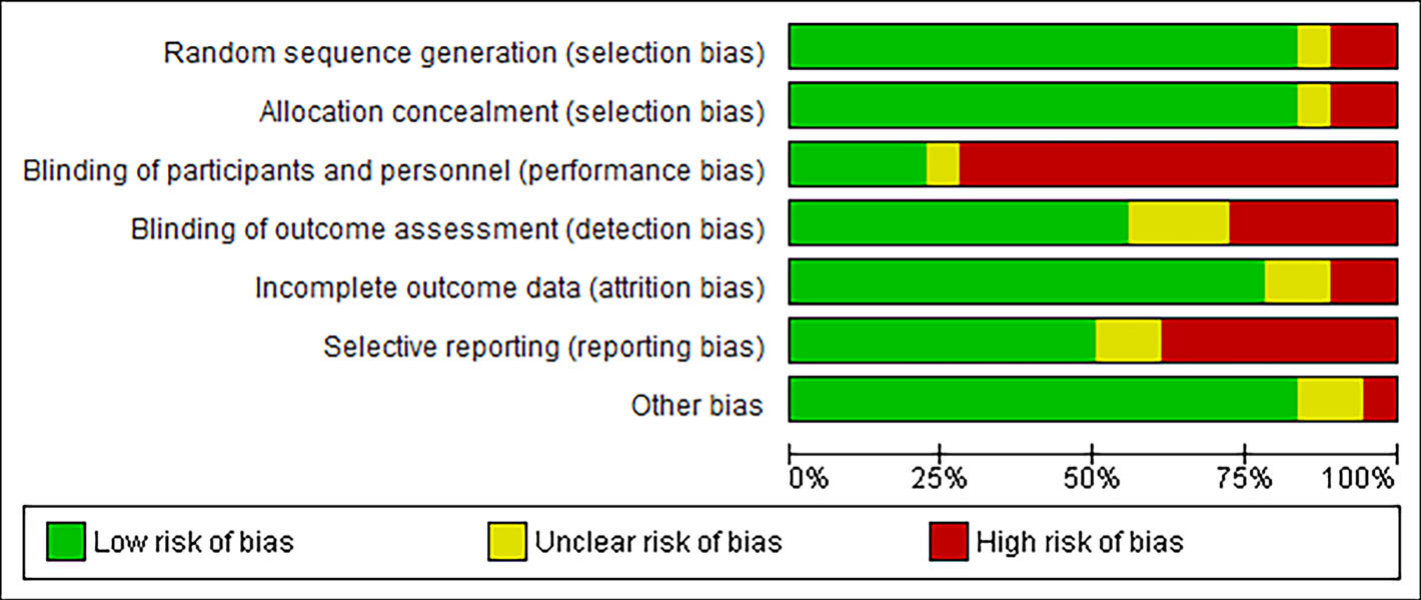
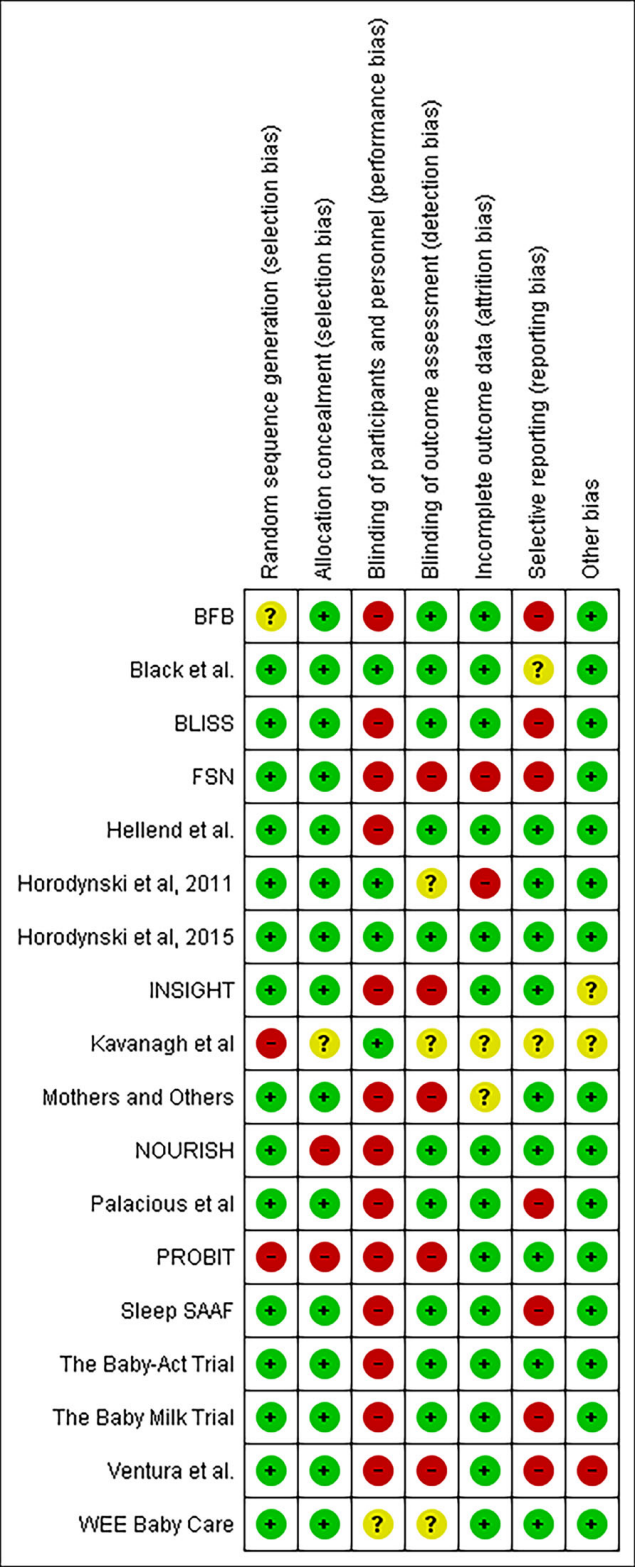

### 3.4 Intervention outcomes and effects

The interventions included in this review were comprised of multifactorial components, which included advice about diet, feeding, and behaviors. Although all interventions included an aspect of responsive feeding, there was considerably heterogeneity in the delivery of intervention components and the outcome measurement. Nine studies reported significant intervention effects on responsive feeding. BLISS found differences in responsive feeding behaviors in favor of the intervention group at 12 but not 24 months.^43^ FSN found significant differences in responsive feeding behavior at 6 and 9 but not at 12 months.^30^ The Home and Videotape intervention reported significant differences at infant age 3 months, the intervention was planned to last for 12 months but no further follow up data was identified.^31^ The INSIGHT study found their responsive parenting intervention led to improvements in responsive feeding at 12 months.^32^, ^33^, ^34^, ^35^ NOURISH reported significant intervention effects for responsive feeding behaviors at infant age 14 months which were sustained for some behaviors at 2, 3.7, and 5 years of age.^40^, ^41^, ^42^ The PROBIT study reported significant differences in responsive feeding in favor of the intervention at 3 months but this was not sustained at 6 or 12 months.^48^ The SMS trial reported that a significantly higher percentage of caregivers in the intervention group stopped feeding when their infants displayed signs of being full.^22^ Mother in the intervention group in the SAAF trial reported more responsive feeding.^24^, ^39^ In The Baby Milk Trial, mothers in the intervention group reported a strengthened attitude to following infant feeding guidelines.^44^, ^45^ Three studies reported no significant changes to responsive feeding post‐intervention^29^, ^38^, ^46^, ^47^; one study did not report responsive feeding behaviors.^36^, ^37^ Five studies were protocols with no published outcomes at the time of reporting^23^, ^25^, ^26^, ^27^, ^28^ (see Table 2).

Four studies reported a significant intervention impact on weight outcomes with one study (FSN) reporting improvements in BMI only,^30^ the NOURISH study reported improvements in BMI and rapid weight gain.^40^, ^41^, ^42^ The PSE intervention reported that infants were at lower risk of rapid weight gain than controls.^38^ Finally, INSIGHT reported a positive intervention effect on weight for age.^32^, ^33^, ^34^, ^35^ However, significant improvements to weight outcomes were not apparent beyond 9 months for FSN,^30^ 14 months for NOURISH and/or not yet reported beyond 6 months (PSE),^38^ or 1 year (INSIGHT).^32^, ^33^, ^34^, ^35^ One study reported that infant growth was significantly higher in the intervention group (*p* < 0.01) contrary to the hypothesis.^29^

### 3.5 Behavior change techniques identified in responsive feeding interventions

A total of 25 BCTs were identified across the 18 interventions in this review (see Table 4 for all identified BCTs). The number of BCTs identified in each reviewed intervention ranged from three^22^, ^43^, ^48^ to 11.^44^, ^45^ The mean number of BCTs identified in the reviewed interventions was six. Five (27%) interventions used >6 BCTs, of which two have reported their findings. The NOURISH intervention (*n* = 8) reported significant findings for responsive feeding and weight.^40^, ^41^, ^42^ The Baby Milk Trial (*n* = 11) reported slower in growth from 0 to 6 months and strengthened maternal attitudes to feeding guidelines in the intervention group but there was no effect on the primary outcome of weight gain to 12 months.^44^, ^45^ The most commonly identified BCT was “Instruction on how to perform a behaviour,” which was identified in 17/18 (94.35%) of the interventions and 8/9 (88%) of the interventions^22^, ^24^, ^30^, ^31^, ^32^, ^33^, ^34^, ^35^, ^39^, ^40^, ^41^, ^42^, ^43^, ^44^, ^45^ reporting higher responsive feeding. This can be seen as the facilitator informing the caregiver how to identify hunger and satiety cues, and how to practice baby‐led weaning.^25^ The second most frequently used BCT was “Adding objects to the environment”; for example, parents were provided with short postcard messages corresponding to the 10 meal principles,^23^ which was identified in 13 (71.5%) of the trials and 7/9 of those reporting higher responsive feeding.^22^, ^30^, ^31^, ^33^, ^34^, ^35^, ^40^, ^41^, ^42^, ^43^, ^48^ Finally, “Demonstration of the behaviour” was used in 6/9 (66%) of trials reporting positive intervention effects for responsive feeding.^24^, ^30^, ^31^, ^32^, ^33^, ^34^, ^35^, ^39^, ^40^, ^41^, ^42^, ^44^, ^45^, ^48^ Seven BCTs were only identified in one trial each (5.5%); these were “Actions planning,”^44^, ^45^ “Reviewing behaviours goals,”^25^ “Feedback on behaviours,”^46^, ^47^ “Feedback on outcome(s) of behaviour,”^44^, ^45^ “Habit formation,”^44^, ^45^ “Social reward,”^28^ and “Restructuring the social environment.”^31^ Of these, only “Restructuring the social environment” was associated with a positive intervention effect on responsive infant feeding in the Home and Videotape intervention.^31^

**TABLE 4:** Presence of BCTs in interventions in included trials.

		BFB (van der Veek^46^; van Vliet^47^)		BLISS (Taylor et al.^43^)	Bottle feeding behaviors (Kavanagh et al.^29^)	FSN (Rosenstock et al.^30^)	Healthy Babies Trial (Horodynski et al.^27^)	Home and Videotape Intervention (Black et al.^31^)	INSIGHT (Paul et al.^35^; Savage et al.^32^, ^34^; Adams et al.^33^)	Mothers and Others (Wasser et al.^36^, ^37^)	NOURISH (Daniels et al.^40^, ^41^, ^42^)	PSE for responsive feeding (Ventura et al.^38^)	PROBIT (Morandi et al.^48^)	Sapere‐sensory education (Helland et al.^23^)	SMS (Palacios et al.^22^)	SAAF (Lavner et al.^24^; Hernandez et al.^39^)	The Baby Act Trial (Campos et al.^25^)	The Baby Milk Trial (Lakshman et al.^44^, ^45^)	Tools for Teen Moms (Horodynski et al.^28^)	Wee Baby Care (Savage et al.^26^)	%
1.1	Goal setting (behavior)	**+**	−	**−**		**+**	−	−	−	−	−	−	−	−	−	−	−	**+**	**+**	−	22.2
1.2	Problem solving	−	−	−		−	**+**	−	**+**	−	−	−	−	−	−	−	−	**+**	**+**	−	22.2
1.4	Action planning	−	−	−		−	−	−	−	−	−	−	−	−	−	−	−	**+**	−	−	5.5
1.5	Review behavior goal(s)	−	−	−		−	−	−	−	−	−	−	−	−	−	−	**+**	−	−	−	5.5
2.1	Monitoring of behavior by other without feedback	−	−	−		−	−	−	−	+	−	**+**	−	−	−	−	−	−	−	−	11.1
2.2	Feedback on behavior	**+**	−	−		−	−	−	−	−	−	−	−	−	−	−	−	−	−	−	5.5
2.3	Self‐monitoring of behavior	−	−	−		−	−	−	−	**+**	**+**	−	−	−	−	−	−	**+**	**+**	**+**	27.7
2.4	Self‐monitoring of outcomes	−	−	−		−	−	−	−	−	−	−	−	−	−	−	−	**+**	−	**+**	11.1
2.7	Feedback on outcomes of behavior	−	−	−		−	−	−	−	−	−	−	−	−	−	−	−	**+**	−	−	5.5
3.1	Social support (unspecified)	−	−	−		−	**+**	−	−	**−**	**+**	−	−	−	**+**	−	−	**+**	**+**	**+**	33.3
4.1	Instruction on how to perform a behavior	**+**	**+**	**+**		**+**	**+**	**+**	**+**	**+**	**+**	**+**	−	**+**	**+**	**+**	**+**	**+**	**+**	**+**	94.3
4.2	Information about antecedents	−	−	−		−	−	−	−	+	−	−	−	−	−	**+**	−	−	−	−	11.1
5.1	Information about health consequences	−	−	−		−	−	−	−	**+**	**+**	−	**+**	**+**	−	**+**	**+**	**+**	−	**+**	44.4
6.1	Demonstration of the behavior	−	−	+		−	**+**	**+**	**+**	**−**	**+**	**+**	**+**	−	−	**+**	**+**	**+**	−	−	52.5
6.2	Social comparison	−	−	−		−	−	**+**	−	+	−	−	−	−	−	−	**+**	−	**+**	−	22.2
6.3	Information about others approval	−	−	−		−	−	−	−	−	−	−	−	−	−	−	**+**	−	**+**	−	11.1
7.1	Prompts/cues	−	−	−		−		−	−	−	−	−	−	−	**+**	−	−	**+**	**+**	−	16.6
8.1	Behavioral practice/rehearsal	**+**	−	−		−	**+**	−	−	**−**	**+**	−	−	**+**	−	**+**	−	−	−	−	27.7
8.3	Habit formation	−	−	−		−	−	−	−	−	−	−	−	−	−	−	−	−	**+**	−	5.5
9.2	Credible source	−	**+**	+		−	−	**+**	−	+	−	−	−	**+**	−	−	**+**	−	−	−	22.2
10.4	Social reward	−	**−**	−		−	−	**−**	−	−	−	−	−	−	−	−	−	−	**+**	−	5.5
12.1	Restructuring the physical environment	−	−	**−**		**+**	**+**	−	−	−	−	−	−	**+**	−	−	−	−	−	−	16.6
12.2	Restructuring the social environment	−	−	−		−	−	**+**	−	−	−	−	−	−	−	−	−	−	−	−	5.5
12.5	Adding objects to the environment	**+**	**+**	**+**		**+**	−	**+**	**+**	**+**	**+**	**+**	**+**	**+**	−	**+**	−	−	−	**+**	76.4
13.1	Identification of self as role model	**+**	−	**−**		**+**	−	−	**+**	**−**	**+**	−	−	**+**	−	−	−	−	−	−	27.7
	Total number of BCTs used in intervention	6	3	4		5	6	6	5	6	8	4	3	7	3	6	7	11	10	6	

*Note*: % = percentage of identified BCTs included in each trial.

Of the four (22%) trials that reported a significant impact of the intervention on an infant's growth and/or weight outcome, 100% included “Adding objects to the environment” and “Instruction on how to perform the behaviour.”^30^, ^32^, ^33^, ^34^, ^35^, ^38^, ^40^, ^41^, ^42^ Three out of the four studies (75%) utilized the BCT “Demonstration of the behaviour”^32^, ^33^, ^34^, ^35^, ^38^, ^40^, ^41^, ^42^ and “Identification of self as role model.”^30^, ^32^, ^33^, ^34^, ^35^, ^40^, ^41^, ^42^

### 3.6 Theories identified in responsive feeding intervention

Of the 18 trials included in this review, 15 (82.5%) mentioned a theory or a model of behavior in the reviewed paper(s).^22^, ^23^, ^24^, ^25^, ^26^, ^27^, ^28^, ^29^, ^31^, ^32^, ^33^, ^34^, ^35^, ^36^, ^37^, ^39^, ^40^, ^41^, ^42^, ^43^, ^44^, ^45^, ^46^, ^47^ Six (44%) of the reviewed interventions were based on a single theory.^22^, ^23^, ^24^, ^26^, ^27^, ^28^, ^29^, ^39^, ^43^, ^46^, ^47^ Over half (66.6%) utilized a theory or predictors to select or develop BCTs.^22^, ^23^, ^24^, ^25^, ^26^, ^27^, ^28^, ^31^, ^33^, ^34^, ^35^, ^39^, ^40^, ^41^, ^42^, ^44^, ^45^, ^46^, ^47^ A total of 14 theories were utilized. The number of theories used to select or develop techniques ranged between zero and four. Three interventions did not use theory,^30^, ^38^, ^48^ 10 interventions used one theory,^22^, ^23^, ^24^, ^26^, ^27^, ^28^, ^29^, ^39^, ^43^, ^46^, ^47^ four interventions used two theories,^31^, ^32^, ^33^, ^34^, ^35^, ^36^, ^37^, ^44^, ^45^ and one intervention used three theories.^25^ The NOURISH intervention used four theories.^40^, ^41^, ^42^ The overall theory score of the trials included ranged from zero to seven, with 7/18 (38%) scoring six or over,^24^, ^26^, ^27^, ^28^, ^31^, ^39^, ^40^, ^41^, ^42^, ^44^, ^45^ 4/18 (22%) scoring five,^22^, ^23^, ^25^, ^32^, ^33^, ^34^, ^35^ and 16.5% scoring zero.^30^, ^38^, ^48^ The most theory was used by the two papers that also used the most BCTs (see above), of which the NOURISH intervention^40^, ^41^, ^42^ reported the most significant effects on responsive feeding and infant growth/weight. The most commonly used theory was the Social Cognitive Theory, which was used in five interventions.^25^, ^31^, ^36^, ^37^, ^40^, ^41^, ^42^, ^44^, ^45^ The Responsive Feeding^32^, ^33^, ^34^, ^35^, ^40^, ^41^, ^42^, ^43^ and the Responsive Parenting theories^24^, ^26^, ^32^, ^33^, ^34^, ^35^, ^39^ were used in three interventions each. The Theory of Planned Behavior was used in two interventions.^27^, ^28^ The following theories were each used once: Cognitive Behavior Theory,^40^, ^41^, ^42^ Ecological Systems Theory,^31^ Experiential Learning Cycle,^29^ Health Self‐Empowerment Theory,^25^ Persuasive Technology Model/Persuasive System Design Model,^25^ Socio‐Ecological Approach,^23^ Stages of Change,^46^, ^47^ and Trans‐Theoretical model^22^ (see Table 5 for all theory and model information).

**TABLE 5:** The presence of theories (Mitchie and Prestwick theory coding method) in the trials.

	BFB (van der Veek^46^; van Vliet^47^)	BLISS (Taylor et al.^43^)	Bottle feeding behaviors (Kavanagh et al.^29^)	FSN (Rosenstock et al.^30^)	Healthy Babies Trial (Horodynski et al.^27^)	Home and Videotape Intervention (Black et al.^31^)	INSIGHT (Paul et al.^35^, Savage et al.^32^, ^34^; Adams et al.^33^)	Mothers and Others (Wasser et al.^37^)	NOURISH (Daniels et al.^40^, ^41^, ^42^)	PSE for responsive feeding (Ventura et al.^38^)	PROBIT (Morandi et al.^48^)	Sapere‐sensory education (Helland et al.^23^)	SMS (Palacios et al.^22^)	SAAF (Lavner et al.^24^; Hernandez et al.^39^)	The Baby Act Trial (Campos et al.^25^)	The Baby Milk Trial (Lakshman et al.^44^, ^45^)	Tools for Teen Moms (Horodynski et al.^28^)	Wee Baby Care (Savage et al.^26^)	%
1. Theory/model of behavior mentioned	+	−	+	−	+	+	+	+	+	−	−	+	+	+	+	+	+	+	77.7
2. Target construct mentioned as predictor of behavior	−	−	+	−	+	+	+	−	+	−	−	+	+	+	−	+	+	+	60.5
3. Intervention based on single theory	+	+	+	−	+	−	−	−	−	−	−	−	+	+	−	−	+	+	44.2
4. Theory/predictors used to select recipients for the intervention	−	−	−	−	−	−	−	−	−	−	−	−	−	−	−	−	−	−	0
5. Theory/predictors used to select/develop techniques	SoC	RF	ELC	−	TPB	EST, SCT	RF, RP	AG, SCT	CBT, AG, RF, SCT	−	‐	SE	TTM	RP	SCT, HS‐ET, PM	SCT, II	TPB	RP	82.5
6. Theory/predictors used to tailor intervention techniques to recipients	+	−	−	−	−	+	−	−	−	+	−	−	+	−	+	−	−	−	27.7
7. All intervention techniques are explicitly linked to at least one theory—relevant/construct/predictor	−	−	−	−	−	−	−	+	+	−	−	−	Unclear	−	−	−	−	−	11.1
8. At least one, but not all, of the intervention techniques are explicitly linked to at least one theory/relevant construct/predictor	+	−	Unclear	−	+	+	+	−	+	−	−	+	+	+	+	+	+	+	66.6
9. Group of techniques are linked to a group of constructs/predictors	−	−	−	−	+	+	+	−	−	−	−	+	−	+	+	+	+	+	49.9
10. All theory‐relevant constructs/predictors are explicitly linked to at least one intervention technique	−	−	−	−	−	−	−	−	+	−	−	−	−	−	−	+	−	−	11.1
11. At least one, but not all, of the theory relevant constructs/predictors are explicitly linked to at least one intervention technique	−	−	−	−	+	+	+	−	+	−	−	+	+	+	−	+	+	+	55.5
12. Theory‐relevant constructs/ predictors are measured	−	−	−	−	+	−	−	−	−	−	−	+	−	−	+	+	+	−	27.7
Total (*n*)	5	2	1	0	8	7	6	2	7	1	0	7	7	7	6	7	8	7	
Items, 7, 8, 9^a^	1	0	0	0	2	2	2	1	3	0	0	2	1	2	2	2	2	2	
Items 9, 10, 11^b^	0	0	0	0	2	2	2	0	3	0	0	2	1	2	1	4	2	2	
Overall theory score^c^	4	2	1	0	6	6	5	2	7	0	0	5	5	6	5	7	6	6	

Abbreviations: AG, anticipatory guidance; CBT, cognitive behavior theory; ELC, experiential learning cycle; EST, ecological systems theory; HS‐ET, health self empowerment theory; II, implementation intentions; PM, persuasive technology model, persuasive system design model; RF, responsive feeding; RP, responsive parenting; SCT, social cognitive theory; SE, socio‐ecological approach; SoC, stages of change; TPB, theory of planned behavior; TTM, trans‐theoretical model.

^a^ Composite score of Items 7, 8, 9 (Item 7 given a weight of +2 for presence, 0 for absence, Items 8 and 9 given a score of +1 for presence, 0 for absence).

^b^ Composite score of Items 9, 10, 11, (Item 10 given a weight of +2 for presence, 0 for absence, Items 9 and 11 given a score of +1 for presence, 0 for absence).

^c^ The overall theory score was calculated by summing Items 3, 4, 5, and 6, with each item weighted as +1. This score was then added to the BCT → theory‐relevant construct score and the theory relevant constructs → BCTs score.

## 4 DISCUSSION

The aim of this review was to identify which BCTs and theories were used in interventions with responsive feeding components for caregivers of children up to 2 years of age to prevent childhood overweight and obesity. A total of 25 BCTs were identified across 16 interventions. Over 70% of the BCTs in the BCTTv1 were not implemented in the interventions included in this review, and many interventions included less than 10% of the BCTs. This is consistent with the findings of a recent systematic review examining the use of BCTs in childhood obesity prevention, which found low reporting of the methods used to identify and apply BCTs in intervention trials.^18^ Although 80% of the trials mentioned theory in their intervention development, the overall theory score was low, with only seven trials scoring 6 or more (out of a possible 8) on the Theory Coding Scheme Michie and Prestwich.^20^. Six studies reported significant intervention effects on responsive feeding and four reported intervention effects on weight outcomes. However, only one intervention reported sustained effects on responsive feeding (NOURISH),^40^, ^41^, ^42^ and no studies reported sustained effects on weight outcome in the included papers.

Inclusion of the BCT, “instruction on how to perform a behaviour” in all but one study aligns with previous reviews that have identified the presence of this BCT in either 100%^17^ or almost 90% of the included intervention.^49^ This review conducted in 2019 also found that 100% of the studies that were considered “effective” included this BCT.^49^ A contributing factor to how much this BCT was used might be that over 50% of the interventions included first time mothers, with the underlying assumption that this group may need more instruction on how to engage in appropriate feeding than mothers with prior experience.^50^ However, providing instruction on how to build the skills to engage in responsive feeding is also important for mothers who had children prior to the intervention as they may need to be shown how to responsively feed if they did not do so previously.^51^, ^52^ While providing instruction and information is important to support appropriate feeding, sustained change requires more than a transactional process of information provision; improving knowledge and skills is only one component of effective behavior change.^13^

The findings that “social support (unspecified)” was identified in 33% of the trials included in this review are unsurprising given that social support is vital for all mothers postpartum, particularly first‐time mothers.^53^ This BCT has been frequently used in other responsive feeding interventions, with one review identifying it as the second most commonly used BCT. Of the six trials that included this BCT in this review, only two included first time mothers only as the participants,^28^, ^40^, ^41^, ^42^ and another trial included participants of which over 50% were first time mothers.^44^, ^45^ Social support for parents, particularly first time mothers, is extremely important given the role of postpartum support in postpartum mental health outcomes such as depression and anxiety.^54^ Further, postpartum mental health issues can impact health behaviors including infant feeding outcomes, such as breastfeeding and perceptions of responsiveness.^55^ Barriers that make accessing social support difficult for all mothers barriers include perceived societal expectations of motherhood and feelings of guilt or shame,^53^ and these are likely the same for mothers seeking support with infant feeding. Including social support components in interventions for first‐time mothers aids in reducing these barriers^53^, ^56^ and potentially increases caregivers likelihood of engaging in appropriate health behaviors for themselves and their infants, including responsive feeding.

Other enablers that enhance parents ability to responsively feed, include education and information, and caregiver attitude to control of feeding.^56^ These factors were addressed and/or enhanced by interventions included in this review as evidenced by identification of BCTs such as “instruction on how to perform the behaviour” and “feedback on the behaviour.”^56^ For instance, one intervention involved videoing participants responsive feeding interactions with their infant, and later using this video to encourage certain behaviors and offer suggestions for improvement.^46^, ^47^ This approach enhanced the amount of information and education the caregivers had regarding their methods, and contributed to the caregiver's positive attitude to the control they have in feeding the infant.

Presence of the BCTs “adding objects to the environment” and “restructuring the environment” in the reviewed interventions could also help to address another factor related to responsive feeding, namely, the influence of the physical environment on caregiver responsiveness.^57^ Both of these BCTs were present in included interventions with the aim of encouraging removal of visual and auditory distractions from the environment such as turning of the television,^40^, ^41^, ^42^ or adding educational resources to educate and encourage caregivers to implement intervention components.^48^ While such individualized BCTs demonstrated some potential beneficial impacts, the inclusion of a larger range of BCTs in future interventions could increase responsive feeding by addressing known barriers and thereby reduce child risk of overweight and obesity. However, it is important to note that different BCTs may have different effects in different populations, particularly given observed differences in infant feeding between populations based on, for instance, ethnicity and socioeconomic status; as such, intervention developers should also consider this when choosing and examining BCTs in complex interventions.

In terms of intervention delivery, this review included a higher percentage of interventions involving a virtual method of delivery, such as via text messages or online software, than has been found in previous research.^22^, ^25^ The virtual delivery of healthcare‐focused preventative interventions has become more common in recent years due to the Covid‐19 pandemic,^58^ and it is likely that the number of interventions that involve a virtual delivery component will continue to increase.^58^ While there was an indication that this method may yield positive intervention effects for responsive feeding,^22^ further research is needed to explore the effectiveness of this approach on behavioral outcomes.

The majority of the interventions in this review included six or more lessons or contacts with the facilitator,^22^, ^25^, ^30^, ^31^, ^32^, ^33^, ^34^, ^35^, ^36^, ^37^, ^40^, ^41^, ^42^, ^43^ and the average intervention delivery time was 9 months with a range of between one^29^ and 12 months.^40^, ^41^, ^42^ This suggests that most trials provided good exposure to the intervention to make a behavioral impact. There is evidence from nutrition‐based education trials involving children and young people, aged 2–19 years of age, that suggests exposure to interventions is more important than duration, with contact time greater than 2 weeks being associated with less successful outcomes.^59^ However, intervention intensity has to be balanced with participant burden because there is also evidence that interventions with over six components are a higher risk of participant attrition.^27^

### 4.1 Strengths and limitations

This provides a systematic review of BCTs in responsive feeding interventions for infants under 2 years of age to prevent childhood obesity. The use of an established taxonomy (BCTTv1) for identification of BCTs and an established theory coding scheme^20^ in the interventions is a primary strength of this review. Use of such systematic and established strategies enabled a thorough and standardized method of systematically identifying distinctive intervention components.^60^ This study conducted a preliminary examination of theory use in responsive feeding interventions, which is important as there are studies that show theory use is linked to intervention effectiveness.^12^ However, our scoring may not adequately represent how theory was used in intervention development due to heterogeneity. A further limitation of this review is the exclusion of studies conducted in non‐OECD countries^61^; this was done to ensure that the findings aid in advising future interventions that aim to prevent childhood overweight and obesity in similar contexts and countries but could reduce the generalizability of findings beyond this context.

## 5 CONCLUSION

In conclusion, this systematic review is the first to report on the use of BCTs and theory in responsive infant‐feeding interventions, delivered by a range of intervention facilitators, with the aim of preventing childhood overweight and obesity. Across the 16 reviewed interventions, BCT and theory use was low. There is a need to systematically develop future childhood obesity prevention interventions, including consideration and application of behavior change approaches and theoretical underpinnings. The findings of the review can inform future intervention development about the most appropriate BCTs to apply. This will increase the opportunity of being able to replicate successful responsive interventions to produce positive results. In doing so, this will aid in reducing the risk of childhood overweight and obesity, and therefore reducing the risk of associated conditions in adulthood.

## CONFLICT OF INTEREST STATEMENT

The authors have no known conflict of interests. Sarah Redsell and Karen Matvienko‐Sikar have received funding from the UK NIHR‐RfPB to develop a responsive feeding intervention. This work is in progress.

## Supporting information


**Data S1.** Risk of Bias.
