# Supplementary material for: Identifying behavior change techniques (BCTs) in responsive feeding interventions to prevent childhood obesity—A systematic review
Source: Obes Rev. 2024 Nov 4;26(2):e13857. doi: 10.1111/obr.13857 (PMC11711079; doi:10.1111/obr.13857)
Supplement: Supplementary file 1 — Data S1. Risk of Bias. [file OBR-26-e13857-s001.docx]

# **Title**

**Identifying Behaviour Change Techniques (BCTs) in responsive feeding interventions to prevent childhood obesity – a systematic review.**

**Supplementary file 1 Risk of Bias**

**INSIGHT**

| Random sequence generation (selection bias) | Low risk |
| --- | --- |
| Allocation concealment (selection bias) | Low risk |
| Blinding of participants, personnel (performance bias) | High risk |
| Blinding of outcome assessment (detection bias) | High risk |
| Incomplete outcome data (attrition bias) | Low risk |
| Selective outcome reporting (reporting bias) | Low risk |
| Other bias | Unclear bias - social desirability bias |
| Summary Risk of bias | Low risk |

Kavanagh et al

| Random sequence generation (selection bias) | High risk |
| --- | --- |
| Allocation concealment (selection bias) | Unclear bias |
| Blinding of participants, personnel (performance bias) | High risk |
| Blinding of outcome assessment (detection bias) | Unclear risk |
| Incomplete outcome data (attrition bias) | Moderate risk |
| Selective outcome reporting (reporting bias) | Unclear risk |
| Other bias | Unclear bias |
| Summary Risk of bias | Moderate risk |

**Black et al.**

| Random sequence generation (selection bias) | Low risk |
| --- | --- |
| Allocation concealment (selection bias) | Low risk |
| Blinding of participants, personnel (performance bias) | Low risk |
| Blinding of outcome assessment (detection bias) | Low risk |
| Incomplete outcome data (attrition bias) | Low risk |
| Selective outcome reporting (reporting bias) | Unclear bias |
| Other bias | Low risk |
| Summary Risk of bias | Low risk |

**The Baby- Act Trial**

| Random sequence generation (selection bias) | Low risk |
| --- | --- |
| Allocation concealment (selection bias) | Low risk |
| Blinding of participants, personnel (performance bias) | High risk |
| Blinding of outcome assessment (detection bias) | Low risk |
| Incomplete outcome data (attrition bias) | Low risk |
| Selective outcome reporting (reporting bias) | Low risk |
| Other bias | Low risk |
| Summary Risk of bias | Moderate risk |

**NOURISH**

| Random sequence generation (selection bias) | Low risk |
| --- | --- |
| Allocation concealment (selection bias) | high risk |
| Blinding of participants, personnel (performance bias) | high risk |
| Blinding of outcome assessment (detection bias) | Low risk |
| Incomplete outcome data (attrition bias) | Low risk |
| Selective outcome reporting (reporting bias) | Low risk |
| Other bias | Low risk |
| Summary Risk of bias | Moderate risk |

**Hellend et al.**

| Random sequence generation (selection bias) | Low bias |
| --- | --- |
| Allocation concealment (selection bias) | Low bias |
| Blinding of participants, personnel (performance bias) | High bias |
| Blinding of outcome assessment (detection bias) | Low bias |
| Incomplete outcome data (attrition bias) | Low bias |
| Selective outcome reporting (reporting bias) | Low bias |
| Other bias | Low bias |
| Summary Risk of bias | Low bias |

**Horodynski et al, 2011**

| Random sequence generation (selection bias) | Low risk |
| --- | --- |
| Allocation concealment (selection bias) | Low risk |
| Blinding of participants, personnel (performance bias) | Low risk |
| Blinding of outcome assessment (detection bias) | Unclear risk |
| Incomplete outcome data (attrition bias) | High risk |
| Selective outcome reporting (reporting bias) | Low risk |
| Other bias | Low risk |
| Summary Risk of bias | Moderate risk |

**Horodynski et al, 2015**

| Random sequence generation (selection bias) | Low risk |
| --- | --- |
| Allocation concealment (selection bias) | Low risk |
| Blinding of participants, personnel (performance bias) | Low risk |
| Blinding of outcome assessment (detection bias) | Low risk |
| Incomplete outcome data (attrition bias) | Low risk |
| Selective outcome reporting (reporting bias) | Low risk |
| Other bias | Low risk |
| Summary Risk of bias | Low risk |

**The Baby Milk Trial**

| Random sequence generation (selection bias) | Low risk |
| --- | --- |
| Allocation concealment (selection bias) | Low risk |
| Blinding of participants, personnel (performance bias) | High risk |
| Blinding of outcome assessment (detection bias) | Low risk |
| Incomplete outcome data (attrition bias) | Low risk |
| Selective outcome reporting (reporting bias) | High risk |
| Other bias | Low risk |
| Summary Risk of bias | Moderate risk |

**Sleep SAAF**

| Random sequence generation (selection bias) | Low risk |
| --- | --- |
| Allocation concealment (selection bias) | Low risk |
| Blinding of participants, personnel (performance bias) | High risk |
| Blinding of outcome assessment (detection bias) | Low risk |
| Incomplete outcome data (attrition bias) | Low risk |
| Selective outcome reporting (reporting bias) | High risk |
| Other bias | Low risk |
| Summary Risk of bias | Moderate risk |

**PROBIT**

| Random sequence generation (selection bias) | High risk |
| --- | --- |
| Allocation concealment (selection bias) | High risk |
| Blinding of participants, personnel (performance bias) | High risk |
| Blinding of outcome assessment (detection bias) | High risk |
| Incomplete outcome data (attrition bias) | Low risk |
| Selective outcome reporting (reporting bias) | Low risk |
| Other bias | Low risk |
| Summary Risk of bias | High risk |

**Palacious et al.**

| Random sequence generation (selection bias) | Low risk |
| --- | --- |
| Allocation concealment (selection bias) | Low risk |
| Blinding of participants, personnel (performance bias) | High risk |
| Blinding of outcome assessment (detection bias) | Low risk |
| Incomplete outcome data (attrition bias) | Low risk |
| Selective outcome reporting (reporting bias) | High risk |
| Other bias | Low risk |
| Summary Risk of bias | Moderate risk |

**FSN**

| Random sequence generation (selection bias) | Low risk |
| --- | --- |
| Allocation concealment (selection bias) | Low risk |
| Blinding of participants, personnel (performance bias) | High risk |
| Blinding of outcome assessment (detection bias) | High risk |
| Incomplete outcome data (attrition bias) | High risk |
| Selective outcome reporting (reporting bias) | High risk |
| Other bias | Low risk |
| Summary Risk of bias | High risk |

**WEE Baby Care**

| Random sequence generation (selection bias) | Low risk |
| --- | --- |
| Allocation concealment (selection bias) | Low risk |
| Blinding of participants, personnel (performance bias) | Unclear risk |
| Blinding of outcome assessment (detection bias) | Unclear risk |
| Incomplete outcome data (attrition bias) | Low risk |
| Selective outcome reporting (reporting bias) | Low risk |
| Other bias | Low risk |
| Summary Risk of bias | Unclear risk |

BLISS

| Random sequence generation (selection bias) | Low risk |
| --- | --- |
| Allocation concealment (selection bias) | Low risk |
| Blinding of participants, personnel (performance bias) | High risk |
| Blinding of outcome assessment (detection bias) | Low risk |
| Incomplete outcome data (attrition bias) | Low risk |
| Selective outcome reporting (reporting bias) | High risk |
| Other bias | Low risk |
| Summary Risk of bias | Moderate risk |

BFB

| Random sequence generation (selection bias) | Unclear risk |
| --- | --- |
| Allocation concealment (selection bias) | Low risk |
| Blinding of participants, personnel (performance bias) | High risk |
| Blinding of outcome assessment (detection bias) | Low risk |
| Incomplete outcome data (attrition bias) | Low risk |
| Selective outcome reporting (reporting bias) | High risk |
| Other bias | Low risk |
| Summary Risk of bias | Moderate risk |

Ventura et al.

| Random sequence generation (selection bias) | Low risk |
| --- | --- |
| Allocation concealment (selection bias) | Low risk |
| Blinding of participants, personnel (performance bias) | High risk |
| Blinding of outcome assessment (detection bias) | High risk |
| Incomplete outcome data (attrition bias) | Low risk |
| Selective outcome reporting (reporting bias) | High risk |
| Other bias | High risk |
| Summary Risk of bias | High risk |

Mothers and Others

| Random sequence generation (selection bias) | Low risk |
| --- | --- |
| Allocation concealment (selection bias) | Low risk |
| Blinding of participants, personnel (performance bias) | High risk |
| Blinding of outcome assessment (detection bias) | High risk |
| Incomplete outcome data (attrition bias) | Moderate risk |
| Selective outcome reporting (reporting bias) | Low risk |
| Other bias | Low risk |
| Summary Risk of bias | Moderate risk |
